# Alpha Satellite DNA in Targeted Drug Therapy for Prostate Cancer

**DOI:** 10.3390/ijms242115585

**Published:** 2023-10-25

**Authors:** Isidoro Feliciello, Đurđica Ugarković

**Affiliations:** 1 Medical School, Department of Clinical Medicine and Surgery, Universiy of Naples Federico II, 80131 Naples, Italy; 2 Department of Molecular Biology, Ruđer Bošković Institute, Bijenička 54, HR-10000 Zagreb, Croatia

**Keywords:** metastatic prostate cancer, satellite DNA, heterochromatin, epigenetics, histone modification, transcription, anti-prostate cancer drugs

## Abstract

Prostate cancer is the most common solid cancer in men and, despite the development of many new therapies, metastatic castration-resistant prostate cancer still remains a deadly disease. Therefore, novel concepts for the treatment of metastatic prostate cancer are needed. In our opinion, the role of the non-coding part of the genome, satellite DNA in particular, has been underestimated in relation to diseases such as cancer. Here, we hypothesise that this part of the genome should be considered as a potential target for the development of new drugs. Specifically, we propose a novel concept directed at the possible treatment of metastatic prostate cancer that is mostly based on epigenetics. Namely, metastatic prostate cancer is characterized by the strongly induced transcription of alpha satellite DNA located in pericentromeric heterochromatin and, according to our hypothesis, the stable controlled transcription of satellite DNA might be important in terms of the control of disease development. This can be primarily achieved through the epigenetic regulation of pericentromeric heterochromatin by using specific enzymes as well as their activators/inhibitors that could act as potential anti-prostate cancer drugs. We believe that our concept is innovative and should be considered in the potential treatment of prostate cancer in combination with other more conventional therapies.

## 1. Introduction

Prostate cancer is the most common solid cancer in men [1]. While surgery and radiotherapy are the dominant treatments for localized diseases, androgen deprivation therapy (ADT), androgen signalling inhibition (ARSI) and chemotherapy are the standard medical treatments for metastatic diseases. While the hormone-sensitive state can last for several years even in the presence of metastatic disease, castration resistance ultimately emerges as a consequence of the strong selective pressure of hormonal therapy on prostate cancer cells [2]. Metastatic castration-resistant prostate cancer represents the final and incurable stage of the disease. Recently, poly (ADP-ribose) polymerase (PARP) inhibitors were introduced in metastatic prostate cancer treatment and they showed clinical benefit for patients with mutations in genes of homologous recombination repair pathways [3]. In addition, prostate-specific membrane antigen (PSMA), which is expressed in prostate cancer cells, can be used to specifically target therapies such as LU-PSMA radioligand therapy [4] or PSMA BiTE—a bispecific CD3 and PSMA antibody that redirects T-cells to PSMA-expressing cells (reviewed in [1,3]). However, despite the development of many new therapies in the last few years, metastatic castration-resistant prostate cancer still remains a deadly disease. It is suggested that the future directions in the management of metastatic prostate cancer should also include the identification of new molecular targets [3].

Satellite DNAs belong to a class of non-coding DNA and, in spite of their presence in relatively large amounts in most eukaryotic genomes, they were considered “junk” DNA for a long time. The reason for that was their association with heterochromatin, which is known to be condensed and inert during most phases of the cell cycle and is impoverished in terms of protein-coding genes [5]. However, results from the last decade show that both satellite DNAs and heterochromatin play important roles in cell physiology, genome stability and evolution [6,7]. Moreover, changes in satellite DNA structure and transcription activity, which lead to the disturbances of heterochromatin structures, are linked to diseases such as cancer as well as senescence and ageing [8]. In this paper, we discuss the importance of satellite DNA transcripts and the dysregulation of transcription associated with disease. We suggest the usage of regulators of satellite transcription as well as satellite transcript modification and stability as possible targets to develop the drugs necessary to control or stabilize satellite DNA transcription and transcripts per se. We specifically address the effects of the major human alpha satellite DNA, the transcription of which was shown to be upregulated in metastatic prostate cancer as well as discuss the control of its transcription and the potential molecules, activators or inhibitors of enzymes involved in satellite DNA transcription regulation. We think that the role of the non-coding part of the genome, satellite DNA in particular, has not yet been sufficiently investigated, especially in relation to diseases such as cancer. Thus, we hypothesise that this part of the genome should be considered in the future as a potential target for the development of new drugs.

## 2. Satellite DNA Characteristics and Regulation

Satellite DNAs are tandemly repeated sequences organized in long Mb-size arrays, which are in most eukaryotic species preferentially located in the (peri)centromeric regions of chromosomes [5]. They are important for the maintenance of the nuclear structure as well as genome integrity and are involved in gene regulation and genome evolution [7,9,10,11,12,13]. In humans, several satellite DNAs are present. Among them, alpha satellite DNA represents the major, most abundant satellite that comprises up to 5% of the whole genome. It is composed of tandemly arranged repeats of the approximate size of 171 bp and is distributed in the (peri)centromeric regions of all chromosomes [14]. Satellite DNA repeats located in (peri)centromeric heterochromatin are actively transcribed, and satellite transcripts are necessary for the formation and maintenance of heterochromatin as well as for centromere function [15,16,17]. In mammals, transcription of satellite DNA proceeds with RNA Pol II in the form of long RNAs being recognized by the endonuclease, Dicer1, which seems to regulate the levels of satellite transcripts [18,19]. In the regulation of the transcription of human alpha satellite DNA, the centromeric protein CENP-B is also involved through the promotion of the binding of the zinc-finger transcriptional regulator (ZFAT), which activates RNA Pol II transcription through the histone modification H4K8Ac [20]. Satellite transcripts form RNA:DNA hybrids which enable the retention of Heterochromatin Protein 1 (HP1) proteins [21] and histone methyltransferases SUV39h1 and SUV39h2. The histone methyltransferases SUV39h1 and SUV39h2 are necessary to establish H3K9me3, which is a major hallmark of heterochromatin [22,23]. Satellite transcripts are additionally enriched with m6A RNA, which is proposed to facilitate their association with pericentromeric heterochromatin [24]. Finally, pericentromeric heterochromatin plays a role in recruiting and/or maintaining cohesin at the centromere to enable proper centromere function and the separation of chromosomes [25].

## 3. Satellite DNA in Cancer

Under physiological conditions, satellite DNA expression is low, as it is temporally and spatially regulated [26,27,28]. However, different pathological conditions activate the transcription of satellite DNAs, and in epithelial cancers such as that of the pancreas, colon, lung, kidney and prostate, a significant increase in satellite DNA transcripts, in particular those belonging to the human satellite II, is detected [29]. Recently, we detected increased levels of major human alpha satellite transcripts in the blood of metastatic prostate cancer patients. We were able to use the levels of alpha satellite RNA to distinguish between different stages of this disease, with metastatic castration-resistant relative to metastatic castration-sensitive [30]. The results revealed that alpha satellite RNA levels could serve as a diagnostic blood biomarker of the metastatic state and particularly of the castration-resistant metastatic type. The increased levels of alpha satellite RNA in the blood of metastatic prostate cancer patients were explained by the transfer of excess satellite RNA from prostate cancer to blood cells by exosomes. Additionally, due to the interaction of exosomes with blood cells, some signalling pathways might be activated that could affect the heterochromatin structure and transcription of satellite sequences located therein [30]. Considering the role of satellite transcripts induced in different types of cancers, numerous studies have shown that they generally promote oncogenic processes using diverse mechanisms, which include cancer therapy resistance [31], the provocation of inflammation [32,33] and tumour cell proliferation [34] and the induction of mutations [35] as well as affecting epigenetic regulators [36] and replication fork stability [37,38].

**Figure 1:**
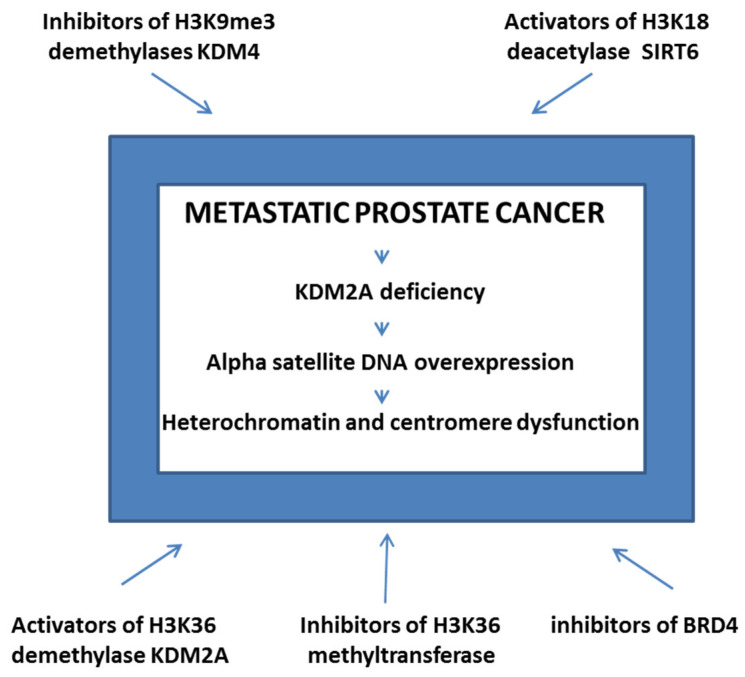
According to our hypothesis, increased levels of alpha satellite RNA, which is characteristic of metastatic prostate cancer, leads to centromere and heterochromatin dysfunction. This causes improper chromosome segregation and genome instability. To restore satellite transcription and genome stability, drugs that affect epigenetic regulators, e.g., enzymes that modulate epigenetic marks on histones within heterochromatin, are proposed. Among the drugs are the following: inhibitors of H3K9me3 demethylases KDM4, activators of H3K18 deacetylase SIRT6, activators of H3K36 demethylase KDM2A, inhibitors of H3K36 methyltransferases and inhibitors of the bromodomain transcriptional co-activator BRD4.

Since the (peri)centromeric regions where satellite DNAs are preferentially located are epigenetically controlled, the lower level of the repressive histone mark, H3K9me3, detected at satellite repeats in cancer cell lines relative to normal cells [39] and global hypomethylation, which is characteristic of cancer cells, can be responsible for aberrant and increased satellite DNA transcriptions [40]. There is also interdependence between DNA methylation and H3K9 methylation in human cells [41]. Specifically, changes in DNA methylation can affect H3K9 methylation and thereby disrupt heterochromatin formation. Reduced H3K9 methylation, on the other hand, can impact nuclear membrane stiffness and integrity [42], which are important for metastasis, or promote tumorigenesis [43]. It is also known that the lysine-specific demethylase 2A (KDM2A), which is specific to H3K36, is downregulated in prostate cancer, and the KDM2A level is negatively correlated with pericentromeric heterochromatin transcription [44]. Therefore, as a result of KDM2A deficiency, increased levels of pericentromeric alpha satellite DNA is observed in prostate cancer, particularly in the metastatic stage [30,44]. Such increased alpha satellite RNA levels affect the pericentromeric heterochromatin structure and centromere function, leading to improper chromosome segregation and genome instability (Figure 1). In addition, the silent state of pericentromeric satellite repeats is also maintained through the action of Sirtuin 6 (SIRT6), which is a member of the Sirtuin deacetylases that deacetylates H3K18ac [45].

## 4. Hypothesis: How to Stabilize Satellite DNA Transcription and Transcripts

Based on the role of satellite transcripts in oncogenic processes, the possible role of alpha satellite transcripts characterized by enhanced levels in metastatic prostate cancer in particular, we propose that the development of drugs used to control or stabilize alpha satellite DNA transcription as well as satellite transcripts could be a potential preventative or therapeutic strategy for metastatic prostate cancer treatment. These drugs are proposed to mostly affect epigenetic regulators, e.g., enzymes that regulate epigenetic marks on histones within heterochromatin (Figure 1). These marks are necessary to stabilize pericentromeric heterochromatin where alpha satellite DNA is located, since the loss of heterochromatin homeostasis is linked to satellite DNA overexpression and promotes genome instability that is a hallmark of cancer. In addition, enzymes that stabilize satellite transcripts per se could also act as potential anti-cancer drug targets.

Among the potential drugs are those that can target histone demethylases and specifically inhibitors of H3K9me3 demethylase, such as KDM4D, which is located in pericentromeric heterochromatin. Since stable H3K9me3 levels seem to be a prerequisite for heterochromatin homeostasis and controlled alpha satellite transcription, these enzymes may be primary drug targets. Other members of KDM4 demethylases, KDM4A-C, also demethylate H3K9me3 and could serve as potential targets. They are overexpressed in several types of cancers [46,47] or are linked to cancer development and maintenance [48,49], and the KDM4 proteins are generally considered as attractive targets for the treatment of cancer patients [50].

In addition, activators of H3K18 deacetylase SIRT6, which are a class of anti-cancer agents, can be used in the same way to inhibit the overexpression of satellite DNA. As previously mentioned, increased H3K18ac levels in pericentromeric heterochromatin is associated with the upregulation of the transcription of alpha satellite DNA located therein [45]. The H3K18ac level is controlled by SIRT6, an NAD+-dependent protein deacylase and mono-ADP-ribosyltransferase of the sirtuin family. In vitro and in vivo studies have indicated the contrasting roles of SIRT6 in cancer; it acts either as a tumour suppressor or promoter in a context-specific manner. Considering the central role of SIRT6 in cellular homeostasis, it has emerged as a target for the development of numerous small-molecule activators and inhibitors possessing therapeutic potential in cancer [51,52].

H3K36 demethylase KDM2A is important for heterochromatin homeostasis and in different types of tumours exhibit either pro-oncogenic or anti-oncogenic effects [53]. Its downregulation in prostate cancer is accompanied by the overexpression of alpha satellite DNA [44], and therefore, it could act as a possible anti-prostate cancer drug target. In addition, inhibitors of H3K36 methyltransferases could also act as potential drugs to control satellite expression in prostate cancer. H3K36 methyltransferases are known to act as cancer drug targets, and their inhibitors are under development [54,55,56]. Besides histone modifying enzymes that regulate the epigenetic state of heterochromatin, endonuclease Dicer1 is also known to control satellite transcript levels. Although the cleavage activity and regulation of Dicer1 needs to be studied more thoroughly, it is known that Dicer-deficient embryonic stem cells show strong proliferation and chromosome segregation defects as well as the increased transcription of centromeric satellite DNA. It was also found that Dicer promotes genome stability via the bromodomain transcriptional co-activator BRD4. Since prostate cancer as well as other cancers are characterized by the upregulated transcription of satellite DNA, BRD4 specific inhibitors can act as promising therapeutic agents with minimal side effects [57].

Finally, an additional potential anti-cancer drug target can be enzymes that stabilize satellite DNA transcripts. Satellite transcripts are stabilized by the methylation of adenosine at position N6 (m6A), and this modification is important for their association with pericentromeric heterochromatin [24]. This modification can be dynamically and reversibly modulated by RNA methylation regulatory proteins, involving m6A methylases and demethylases. It was shown that deregulated m6A methylation participated in numerous pathologic processes including tumorigenesis, progression and metastasis [58]. m6A demethylases fat mass and obesity-associated protein (FTO) and alkB homolog 5 (ALKBH5) were shown to play oncogenic roles in various cancers, and in vitro studies confirmed that FTO-specific inhibitors exhibited anti-tumour effects. This suggests that FTO and ALKBH5 demethylases can act as therapeutic targets and their inhibitors as potential m6A-targeting anti-cancer drugs [59,60]. It is also important to mention that a new, simple method was developed to specifically measure the transcription of alpha satellite DNA [61] and enables the monitoring of the levels of transcripts during therapeutic protocols e.g., tracking the effects of therapy on alpha satellite expressions.

## 5. Conclusions

Prostate cancer is a common disease and while the localized form of the disease is curable, the metastatic hormone-sensitive disease lasts several years until castration resistance emerges as a consequence of the strong selective pressure of hormonal therapy. Metastatic castration-resistant prostate cancer represents the final and incurable stage of the disease. In the last few years, many new therapies are under development, and it is suggested that future directions in the management of metastatic prostate cancer should include the identification of new molecular targets [3]. This article proposes a novel concept directed at the possible treatment of metastatic prostate cancer, which is mostly based on regulating the transcription of non-coding DNA and satellite DNA in particular. Namely, metastatic prostate cancer is characterized by the induced transcription of alpha satellite DNA, which is located in pericentromeric heterochromatin. According to our hypothesis, the stabilization of alpha satellite DNA transcription and satellite transcripts is a potential preventative or therapeutic strategy for metastatic prostate cancer treatment as well as the control of disease development. This control could be mostly achieved through epigenetic regulators of pericentromeric heterochromatin. We first highlight the major epigenetic modifications of pericentromeric heterochromatin that shape its structure and are important for the stable transcription of the satellite DNA located therein. We then discuss the enzymes responsible for such modifications as well as their activators/inhibitors that could act as potential anti-cancer drugs. We believe that our concept is innovative and should be considered as a novel approach for possible treatment of prostate cancer in combination with other more conventional therapies.

## Data Availability

Not applicable.
